# A Unique Population in a Unique Area: The Alcon Blue Butterfly and Its Specific Parasitoid in the Białowieża Forest

**DOI:** 10.3390/insects11100687

**Published:** 2020-10-12

**Authors:** Izabela Dziekańska, Piotr Nowicki, Ewa Pirożnikow, Marcin Sielezniew

**Affiliations:** 1 Division of Molecular Biology, Faculty of Biology, University of Bialystok, Ciołkowskiego 1J, 15-245 Białystok, Poland; 2 Institute of Environmental Sciences, Jagiellonian University, Gronostajowa 7, 30-387 Kraków, Poland; piotr.nowicki@uj.edu.pl; 3 Institute of Forest Sciences, Bialystok University of Technology, Wiejska 45 E, 15-351 Białystok, Poland; e.piroznikow@pb.edu.pl; 4 Laboratory of Insect Evolutionary Biology and Ecology, Faculty of Biology, University of Bialystok, Ciołkowskiego 1J, 15-245 Białystok, Poland; marcins@uwb.edu.pl

**Keywords:** adult demography, *Ichneumon eumerus*, *Maculinea alcon*, *Myrmica scabrinodis*, host-ant specificity, life span, mark–recapture, population size, temporal fragmentation

## Abstract

**Simple Summary:**

Caterpillars of the Alcon blue butterfly *Phengaris alcon* feed initially inside flowerheads of *Gentiana* plants but complete their development as ‘cuckoos’ in nests of *Myrmica* ants being fed by workers. Social parasitism protects larvae from most natural enemies and only specialized ichneumon wasps are able to infiltrate host colonies and parasitize them. Across its range *P. alcon* forms different ecotypes adapted to specific ants and plants. Complicated ecological requirements make the butterfly a very local and threatened species and sensitive to environmental changes. We investigated an isolated and previously unknown population in the high nature value area, i.e., the Białowieża Forest (NE Poland). Using the marking technique we estimated the seasonal number of adults at 1460 individuals and the density (850/ha) showed to be the highest among all hygrophilous populations studied so far. Premature *P. alcon* were found exclusively in nests of *M. scabrinodis* and from as many as 75.5% pupae *Ichneumon* cf. *eumerus* wasps were reared. The exceptional abundance of both *P. alcon* and its parasitoid (its population could be estimated at about 4500 adults) can be explained by a high density of nests host ants in vicinity of host plants. This unique system deserves special conservation care.

**Abstract:**

Caterpillars of the Alcon blue butterfly *Phengaris alcon* are initially endophytic and feed inside the flowerheads of *Gentiana* plants, but complete their development as social parasites in the nests of *Myrmica* ants, where they are fed by workers. Its specific and complicated ecological requirements make *P. alcon* a very local, threatened species, sensitive to environmental changes. We investigated an isolated and previously unknown population in an area of high nature value—the Białowieża Forest (NE Poland). Using the mark–release–recapture method we estimated the seasonal number of adults at 1460 individuals, and their density (850/ha) was the highest among all populations using *G. pneumonanthe* studied so far. The site is also unique due to the presence of the specific parasitoid *Ichneumon* cf. *eumerus,* and parasitoids are considered the ultimate indicators of the biodiversity of *Phengaris* systems. Since 75.5% of *P. alcon* pupae were infested we could estimate the seasonal population of adult wasps at about 4500 individuals. The high abundance of both *P. alcon* and its parasitoid may be explained by favorable habitat characteristics, i.e., the strong presence of host plants and the high density of nests of *Myrmica scabrinodis*, which is the only local host ant of the butterfly.

## 1. Introduction

European representatives of the genus *Phengaris* Doherty (Lepidoptera, Lycaenidae), which is a senior synonym of *Maculinea* Van Eecke [1], are considered as flagship priorities in butterfly conservation, along with Queen Alexandra’s birdwing of Papua New Guinea, and the Mexican overwintering sites of monarchs [2]. All four European species are threatened on a continental scale [3] and three of them are also listed in the Annexes of the Habitat Directive. Large blues attract the attention not only of conservationists but also scientists due to their complicated and peculiar life history related to obligatory myrmecophily [4,5,6].

In contrast to most other butterflies they require two different resources to complete their development. Caterpillars are initially endophytic, feeding on specific plants, but in the fourth (final) instar they become social parasites of red ants (*Myrmica* Latr.). Infiltration of host colonies is enabled by sophisticated adaptations, including chemical and acoustical mimicry [7,8,9]. Three of the four European species exploit ants as predators feeding on their brood, while the Alcon blue *Phengaris alcon* (Denis and Schiffermüller, 1775) is mostly fed directly by ants through trophalaxis or by insect prey. This sophisticated strategy is believed to be the most advanced and also the most effective, i.e., more ‘cuckoo’ larvae can develop in a single colony compared to predatory species [10].

*Phengaris alcon* shows considerable ecological variation across its range, being locally adapted to different larval food plants and host ants. Populations inhabiting contrasting biotopes even used to be regarded as different species, e.g., those dependent on *Gentiana cruciata,* which grows mostly on xerothermal calcalerous grasslands, were commonly separated out as *P. rebeli,* while the nominative occurred on wet meadows with *Gentiana pneumonanthe*. However genetic studies showed that this approach was unreasonable, and moreover the number of larval food plants used is even higher [11,12,13,14,15].

In the European lowlands the most common ecotype is that dependent on *G. pneumonanthe.* Quite often it exists in very small and spatially isolated local populations, however, large metapopulation systems with many connected populations are also known [16,17,18,19,20,21,22]. Two host ant races could be distinguished in Europe, i.e., north-western populations depending on *Myrmica rubra* and *Myrmica ruginodis,* while the remaining ones mostly exploit the nests of *Myrmica scabrinodis* [23].

In 2016 we discovered a large population in an area of high nature value—the Białowieża Forest (NE Poland). The area is believed to be the best preserved forest complex in temperate lowland Europe. It is known as the most important refuge for primeval forest entomofauna, and saproxylic species (especially beetles) are considered the most valuable component [24]. Less common knowledge is that the Białowieża Forest is also considered one of the best explored and richest areas in Poland as far as Lepidoptera are concerned [25,26,27,28], being one of the prime butterfly areas [29]. A total number of 110 species have been recorded from the Białowieża Forest so far, i.e., more than 2/3 of Polish fauna, but there are no data concerning the presence of *P. alcon* there.

The aim of the present study was to estimate the demographic parameters of the adult population at this isolated site. We also identified host ants and assessed density in the vicinity of food plants as one of the key parameters determining the habitat quality of the butterfly. Finally we studied the infestation of *P. alcon* pupae by *Ichneumon* cf. *eumerus,* which was recorded during a preliminary survey. This rare and specific parasitoid attacks butterfly larvae when they are in the socially parasitic part of their development [30]. Simultaneous studies performed in the same season enabled us to estimate the seasonal population size both of adult butterflies and ichneumon wasps, for the first time.

## 2. Materials and Methods

### 2.1. Study Site

**Figure 1:**
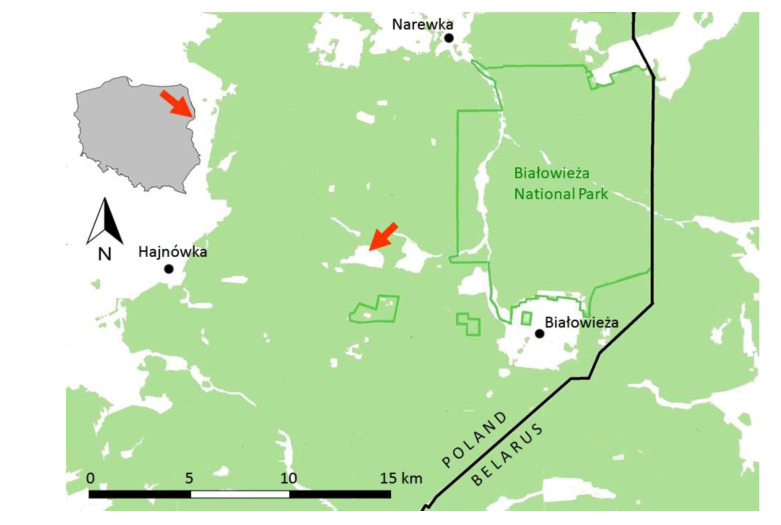
Outline map of the Białowieża Forest (NE Poland) with the location of the study site (red arrow). The color green represents area covered by forest.

**Figure 2:**
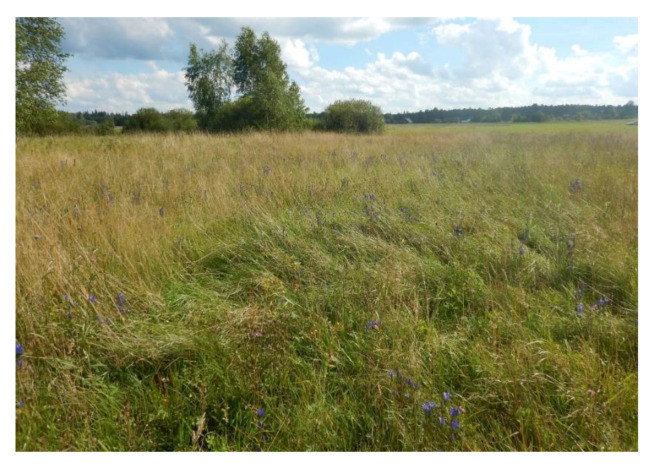
The study site of *Phengaris alcon* in the Białowieża Forest.

We conducted our study on an isolated site of *P. alcon* near the village of Budy (52°44′10″ N, 23°44′7″ E; 150 m above sea level), situated in one of the large clearings in the Białowieża Forest and surrounded by compact forest stands (Figure 1). This open area surrounded by forest had an area of about 120 ha and nearly 90% of it was covered by grasslands and fallow land. The area with *Gentiana pneumonanthe* (Figure 2) covered only 1.71 ha and encompassed parts of six plots of land differing with regard to management. Three of them were irregularly mown once a year and on three others no signs of recent (i.e., within at least the last five years) management could be seen. In the past the whole area was probably extensively mown.

The biotope of the butterfly could be classified as a mesotrophic fen-meadow community of *Molinietum caeruleae* W. Koch 1926 (*Molinietum medioeuropaeum* W. Koch 1926; ‘litter meadows’), with somewhat impoverished species composition. The phytocoenoses was dominated by grasses, i.e., mainly *Phleum pratense*, *Festuca rubra* and *Agrostis capillaris,* and with a smaller proportion of *Deschampsia caespitosa* and *Briza media*. In some patches the development of aggregations of *Calamagrostis epigejos* was observed. *Juncus effusus* and *Juncus conglomeratus* could be also mentioned, among other monocots. The meadows were characterized by a high species richness of dicotyledonous plants, and the most common were *Centaurea jacea*, *Lathyrus pratensis*, *Viola canina*, *Potentilla erecta* and *Lychnis flos-cuculi,* and *G. pneumonanthe* in some parts. Local aggregations of *Betonica officinalis, Succisa pratensis* and *Rhinanthus serotinus* were also remarkable. In wetter places *Lysimachia vulgaris* and *Filipendula ulmaria* were also present.

The abundance of the host plant was quite high, an estimated 3000 individuals at least, based on counts of individuals present in six random plots (50–100 m^2^). Only a few single plants were localized outside the study area.

### 2.2. Studies of Adult Demography

**Figure 3:**
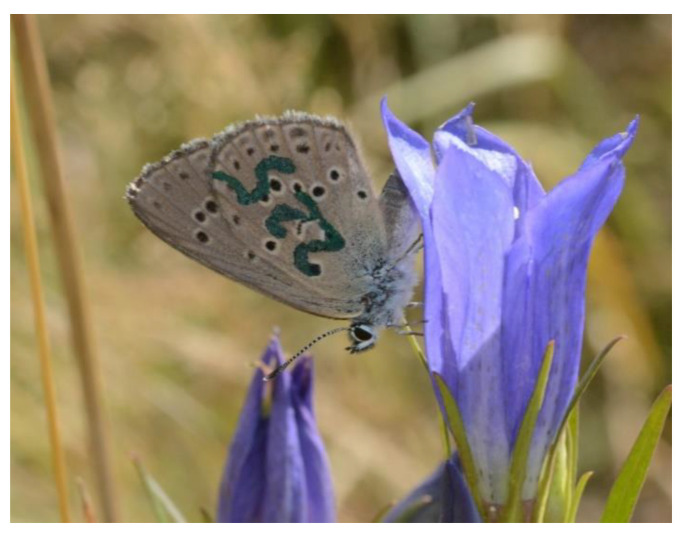
A marked individual of *Phengaris alcon* on a flower of *Gentiana pneumonanthe.*

The study population was monitored every year since its discovery, and in every season adults were observed numerously at the site. A mark–release–recapture (MRR) study to estimate population size and adult life span was conducted in 2019, when we sampled the population on 11 occasions between 19 July and 12 August. The sampling covered almost the entire flight period of the focal species, i.e., it was started on the second or third day of the emergence of the first adults and the site was visited every 2–5 days if the weather was favorable (i.e., sunny and not very windy), between 10 am and 5 pm. Two people spent 2–3 h on the site during each sampling day. The end of the flight period was estimated at around 16 August, so its total duration was ca. 30 days. Butterflies were captured with an entomological net, marked on the underside of their hind-wings with unique identity numbers (Figure 3) using a fine-tipped waterproof pen, and then immediately released at the place of capture. Date, time and patch number and the sex and ID number of each butterfly were recorded.

We analyzed our data with the Jolly-Seber model [31] using Program MARK, version 8.0 [32]. The model represents a well-established standard for estimating the population size in open populations, and it has been frequently applied in butterfly studies [33,34,35]. Based on the lowest value of the Akaike information criterion corrected for small sample size (AIC_c_) [36], the best-performing model variant was *φ* (.) *p* (*s + t*) *B* (s*t), i.e., the model assuming a constant (and equal for both sexes) survival rate (*φ*), but sex-dependent and freely time-varying (thus differing between capture days) recruitments of new individuals into the population (*B*), and sex-dependent and time-varying capture probabilities (*p*) with a constant difference between sexes. We thus used this model to obtain the estimates of daily numbers of males and females as well as their seasonal population sizes. Subsequently, we estimated the total population size as the sum of the male and female population sizes. For comparative purposes we also derived mean capture probabilities for males and females across the entire season and mean adult life span, estimated from the survival rate as *e* = (1 − *ϕ*)^−1^ − 0.5 [37]. Besides, we calculated the temporal fragmentation index, i.e., ratio of the flight period length to the adult life span, which is considered an indicator of species vulnerability [38].

### 2.3. Studies of Premature Individuals in Ant Nests

In late August 2018, patches with larval food plants bearing eggs/eggshells of *P. alcon* were marked with GPS, and additionally using wooden tags. In late June and early July 2019 we searched for *Myrmica* ants in those patches in a radius of 1 m from plants, i.e., certainly within the foraging zone of *Myrmica* workers [39]. All nests found were very carefully opened and examined for the presence of *P. alcon*. It is known that full-grown larvae are carried by workers to upper chambers during the day and that pupation takes place there as well [40], therefore there was no necessity for the excavation and destruction of colonies. For conservational reasons (the sensitivity of an isolated population) we did not try to find all the premature individuals, which could be present in deeper chambers if a colony was infested. Nests in which we could not find any larvae/pupae were searched more thoroughly. Finally we covered the nests and restored the arrangement of the surrounding vegetation as exactly as possible to minimize the impact of our investigation.

When we found prepupae and/or pupae, they were put into plastic containers with some wet moss and transferred to the lab. They were maintained there at room temperature until adult butterflies or wasps emerged. The proportion of parasitized pupae enabled us to estimate the population size of adult parasitoids, based on the estimation of the butterfly population derived from the MRR study.

Additionally, 50 randomly chosen squares (1 m^2^ each) were surveyed to estimate the average density of *Myrmica* colonies in the vicinity of *G. pneumonanthe* plants. Ants were preliminarily identified in the field with hand lenses, but voucher samples of 5–10 workers were collected to confirm this determination in the laboratory, according to Czechowski et al. [41].

## 3. Results

### 3.1. Adult Demography

We captured and marked a total of 854 individuals (505 males and 349 females). Only 154 of them (18%) were recaptured. The proportion of recaptured males and females was almost identical (17.8% and 18.3% respectively). The maximum number of recaptures on different days was four for males and three for females. Most of the capture events occurred in more sheltered places, surrounded by trees and shrubs.

The mean number of days between first and last captures was 2.63 for males and 2.86 for females respectively, whereas the maximum duration between captures of an individual reached six days for both sexes. The daily survival rate obtained with the Jolly-Seber model was 0.558 ± 0.021 (95% Confidence Interval [CI]: 0.517–0.579), which corresponds to the estimated adult life span of 1.76 ± 0.11 days (95% CI: 1.57–1.99 days). Taking into consideration the estimated flight period (ca. 30 days) the temporal fragmentation index was calculated at 17.05.

**Figure 4:**
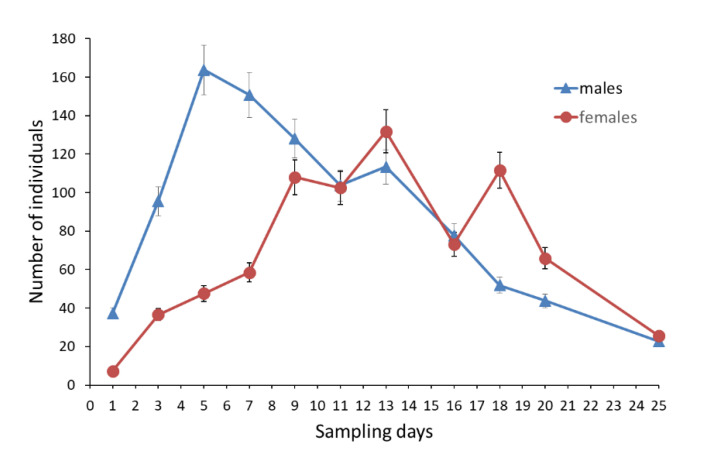
Dynamics of daily numbers of males and females throughout the flight period based on Jolly-Seber model estimates. Error bars represent SEs.

The mean capture probability was slightly higher in males (0.617 ± 0.078; 95% CI: 0.457–0.755) than in females (0.547 ± 0.069; 95% CI: 0.412–0.675). In the first week of sampling, a clear dominance of males was observed with the peak of abundance on July 23rd (164 ± 12.8). The highest number of females was estimated eight days later (132 ± 11.2). Generally the dominance of females in the second half of the flight period was less evident compared to that previously observed for males (Figure 4).

The seasonal population size was estimated at 1460 adults (95% CI: 1358–1582), including 793 males (95% CI: 722–886) and 667 females (95% CI: 599–753) giving a slightly male-biased sex ratio (1.19:1). The density was estimated as 854 adults per hectare.

### 3.2. Host Ants and Parasitoids

**Figure 5:**
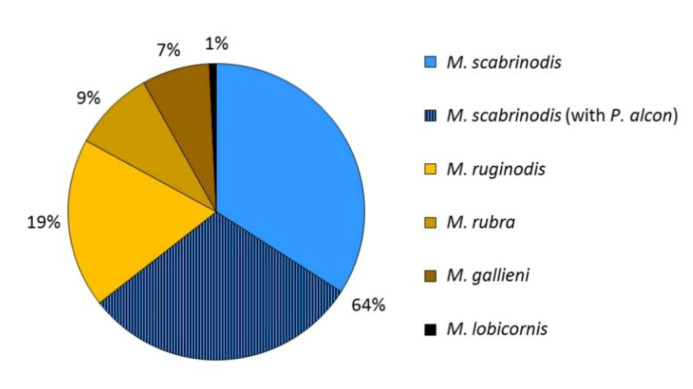
*Myrmica* ant species composition at the site of *Phengaris alcon* in the Białowieża Forest and proportion of *Myrmica scabrinodis* nests infested by the butterfly.

A total number of 135 colonies of *Myrmica* ants was found and inspected. The most common species was *M. scabrinodis* Nyl. (87 nests) and the other four species recorded there were: *M. ruginodis* Nyl. (25), *M. rubra* (L.) (12), *M. gallieni* Bondr. (10) and *M. lobicornis* Nyl. (1) (Figure 5).

Premature *P. alcon* were found exclusively in colonies of *M. scabrinodis* (Figure 5) and the difference in the rate of parasitism between this species and the three other most common species was significant according to the Fisher exact test: *M. scabrinodis/M. ruginodis* (*p* < 0.00001), *M. scabrinodis/M. rubra* (*p* = 0.0003) and *M. scabrinodis/M. gallieni* (*p* = 0.0013).

We found a total number of 94 premature individuals: larvae, prepupae and pupae (1–13 in individual nests and 2.29 on average). However, as far as infested colonies are concerned our search was restricted to surface chambers so as not to disturb them excessively and to minimize our impact on the population. Therefore, it is possible that further larvae were present there.

**Figure 6:**
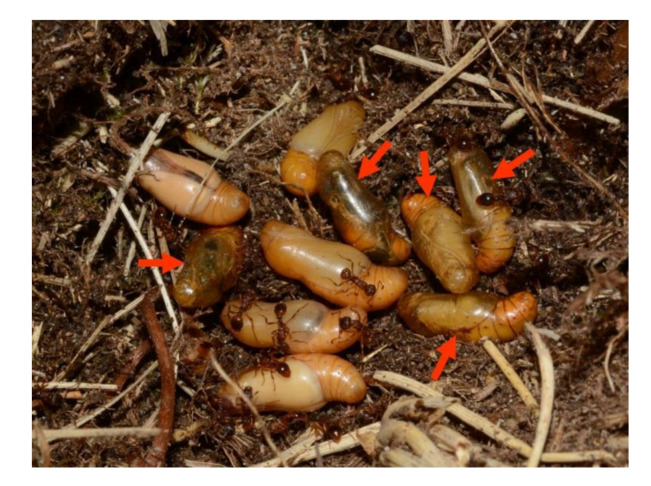
Parasitized (marked with arrows) and unparasitized pupae of *Phengaris alcon* in a nest of *Myrmica scabrinodis.*

**Figure 7:**
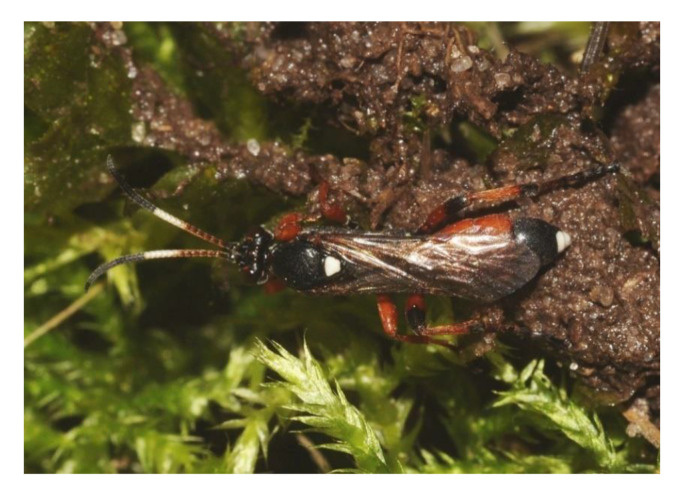
A female *Ichneumon* cf. *eumerus* reared from a pupa of *Phengaris alcon.*

The larvae we found were left with their hosts, but prepupae and pupae (Figure 6) were collected and kept in captivity to assess the parasitization rate. A total number of 49 individuals were collected from 27 different nests, and 37 pupae (75.51%) from 21 nests (1–6 parasitized pupae per a nest) were finally shown to be parasitized by *Ichneumon* cf. *eumerus* Wesmael, 1857 (Figure 7). This indicates that the population of adult wasps could be three times more numerous than the population of adult butterflies. Based on this rate and an estimation of the adult population size of *P. alcon* (1460)*,* the seasonal number of adult wasps could be estimated at ca. 4500 individuals.

The mean density of *Myrmica* nests near gentians was 1.86/m^2^ and density of colonies of the only recorded host ant, i.e., *M. scabrinodis,* was estimated at 1.34/m^2^. Up to four nests were found in a single 1 m^2^ square.

## 4. Discussion

### 4.1. The Alcon Blue Butterfly in the Białowieża Forest

Surprisingly *P. alcon* has never been recorded in the Białowieża Forest before, including the Belarussian part of the area [42]. We hypothesized that the presently studied site was simply overlooked in the past due to its small area and inconspicuous surroundings. Recent colonization is not likely, taking into consideration the very specific and complex habitat requirements of *P. alcon* and restricted colonization abilities [16]. Some butterfly species colonized the area of the Białowieża Forest recently, following a range expansion, as, e.g., observed for the generalistic *Melanargia galathea*. There are also records concerning habitat specialists, namely *Lycaena helle,* which became extinct at a historical site, although several populations have recently been discovered in other parts of the Białowieża Forest. It is suggested that it could have colonized some sites as a consequence of actions aimed at the restoration of meadows in river valleys [43]. However the larval food plant of *L. helle* is relatively widespread compared to *G. pneumonanthe,* which is rare, local and declining in the Białowieża Forest [44]. Moreover species like *P. alcon,* due to its absence in Annex II of the Habitat Directive, are not included in management plans for Natura 2000 sites, and are therefore neglected by most inventory and monitoring activities.

### 4.2. Adult Demography

The most unique trait of the presently studied population is the density of adults, which is the highest of all populations of *Phengaris alcon* using *Gentiana pneumonanthe* studied so far with the MRR method (Table 1). If we also consider the xerothermophilous form of *P. alcon* dependent on *G. cruciata* (formerly known as *P. rebeli*) only one single German population reached a higher abundance per hectare [45]. However it occupied an area that was a few times smaller, and the overall number of adults was lower. Another method of estimation of adult populations is based on egg counts but the maximum result for hygrophilous populations was clearly lower [46].

The high density of adults reflects two favorable habitat characteristics, i.e., (i) high and relatively even coverage by initial larval food plants and (ii) the high density of host ants. If we compare our results with data concerning other *Phengaris* species, especially for *P. teleius* and *P. nausithous,* even higher densities (ca 1000 adults per hectare) are found on some sites [19,37]. One has to keep in mind that the larval food plant of congeneric species (*Sanguisorba officinalis*) is often abundant and evenly distributed in favorable biotopes, in contrast to *G. pneumonanthe,* which usually forms local clusters. Therefore the importance of host plant availability in determining of habitat quality may vary between species [19].

The estimated life span of adults of *P. alcon* in our study (1.7 days) was relatively low but not exceptional if compared with available data [20,22,47,48,51,52], and *Phengaris* butterflies are generally considered short-lived insects (see [38]). Their short life expectancy is compensated for by high fecundity, and in the case of *P. alcon* a female can lay 19 eggs/h and 80–100 eggs per day [51].

Interestingly, the estimated life span (4.9 days) of another isolated population studied in the same region was nearly three times longer [22]. This discrepancy could be related to the high density of adults at the presently investigated site, which could trigger emigration [34] and therefore affect the recorded residency time. The population in the Białowieża Forest was clearly isolated, but at the same time the occupied patch of habitat was situated in an open area of grassland and fallow land without clear barriers and such conditions favor dispersal [17]. As a consequence many individuals could leave the site relatively freely. In contrast, the previously studied population was very small and its site was surrounded by a clearly distinct biotope of wetlands with tall vegetation of sedges and *Phragmites* [22].

The calculated value of the temporal fragmentation index was then relatively high and typical for a species of conservation concern (see [38] for a review), suggesting that the population could be under threat. However, taking into consideration the high density of adults and weak protandry we could assume that the chance for individuals to find a mate was high for most of the flight period and the effective population was not reduced.

The slightly higher capture probability of males that we recorded is not surprising taking into consideration the brighter colouration of this sex and its patrolling behavior. Females are less conspicuous when they are flying low and looking for larval food plants or ovipositing on gentians, which are frequently still not flowering. Tall grasses in some parts of the habitat made the detection of females even harder. On the other hand the higher catchability of patrolling males was also observed for the congeneric *P. arion,* which in contrast shows weak sexual dimorphism and usually occurs in lower vegetation [35]. This may suggests that flight activity is the most important factor influencing probability of encountering a *Phengaris* of a given sex.

### 4.3. Host Ants and Parasitoids

Host ant specificity turned out to be typical for this part of the European range [23], and for Poland in particular. *Myrmica scabrinodis* is the only host ant of *P. alcon* on the vast majority of Polish sites where *G. pneumonanthe* is the larval food plant [53]. The exceptions are habitats where *Myrmica vandeli* also co-occurs, but this ant is a closely related species and moreover suspected to be a temporary social parasite of *M. scabrinodis* [54]. Among hundreds of nests searched across the country there was just a single case of the use of an untypical host (i.e., *Myrmica schencki*) by the hygrophilous ecotype [55]. A more complicated pattern of host use is observed in the case of populations dependent on *G. cruciata* [23].

The density of host nests was relatively high, i.e., >1 colony of *M. scabrinodis* was recorded per square meter on average in the vicinity of gentians. There is an ongoing debate over whether females of *P. alcon* are able to detect their host, and the results are ambiguous [56,57,58]. In the case of the presently studied site this potential ability seems to be less important, taking into consideration the high availability of hosts. Moreover it was found that *P. alcon* may switch between ant nests during its development, e.g., a weak colony with the butterfly’s caterpillars inside raided by a stronger colony [59]. In that case a high density of host colonies increases the probability that readopted caterpillars will be moved to a nest where they will be able to complete their development.

Not only its high abundance makes the *P. alcon* population from the Białowieża Forest unique and deserving of conservational concern. The presence of a specific parasitoid was detected and its impact on the population is likely to be significant taking into consideration that as many as three quarters of the pupae were infested. This means that the adult population of this wasp is around three times more numerous compared to adult butterflies. Parasitoids are important factors influencing the population size of butterflies. However, socially parasitic *Phengaris* butterflies are known as regular hosts of specific ichneumonid parasitoids of the subfamily Ichneumoninae, almost exclusively [60]. Three species are attacked when they are still in the flowerheads of larval food plants. *Phengaris arion* is infested by *Neotypus coreensis* but data on its occurrence are very rare [61]. A more widespread species is *Neotypus melanocephalus* Gmelin, 1790, a specific parasitoid of *Phengaris nausithous* (Bergsträsser, 1779)*,* which may be quite common at some localities [62]. This wasp can also occasionally parasitize *Phengaris teleius* (Bergsträsser, 1779) [63], which in turn can be a rare host of *Ichneumon fulvicornis* Gravenhorst 1829 and *I. eumerus* Wesmael 1857 [60].

As far as *I. eumerus* is concerned it is known mostly as a spectacular and famous specific parasitoid of *P. alcon.* Females localize their host only in ant colonies and produce specific allomones, causing fights among workers to parasitize butterfly caterpillars with impunity [30,64]. There are anecdotal records of *P. alcon* parasitized by *Ichneumon balteatus* Wesmael 1845, known as a parasitoid of some other unrelated butterfly species [65].

Data on the occurrence of populations of *P. alcon* infected by parasitoids are scarce. In Poland hygrophilous populations have been recorded so far only from a single region in the south situated about 300 km from the presently studied site. The parasitization rate of pupae found in nests of *M. scabrinodis* and *M. vandeli* was also high there, estimated at 62% [54]. Moreover there is a record from the site of the xerothermophilous form using *G. cruciata* where just a single nest with infested pupae was recorded among many dozens surveyed [66].

In addition, parasitoids of *P. alcon* have been reported from the Spanish Pyreenes [30], Hungary [67], Portugal [68] and from the Austrian Alps [69], where quantitative data concerning the parasitization rate are available—60% and 77.6% of pupae of *P. alcon rebeli* and *P. alcon* X were infested by *I.* cf *eumerus* respectively. It should be mentioned here that there are some uncertainties concerning the identity of *Ichneumon* wasps parasitizing different ecotypes/subspecies of *P. alcon* [60], and this is why we identified the reared wasps as *I*. cf *eumerus*. Genetic studies are desirable, to look for possibilities of cryptic speciation among populations adapted to different biotopes.

### 4.4. Implications for Conservation

Despite the positive features discussed above, the investigated population may be at risk of extinction given factors including isolation, successional changes and unpredictability of management practices. Butterflies requiring grasslands have suffered a severe decline in the Białowieża Forest and some species (including habitat directive species, i.e., *Parnassius mnemosyne, Colias myrmidone, Polyommatus eros, Phengaris arion, Coenonympha hero* and *Coenonympha oedippus)* have become extinct at least in the Polish part [28]. They were mainly affected by intensification of management or abandonment. Many open areas have become overgrown and invasive plants are also a real problem.

Our one-year study does not enable evaluation of the long-term trend of the investigated population. However, it is worth noting that populations of *Phengaris* species are considered as relatively stable compared to other butterflies [70]. There is no doubt, however, that certain conditions are not stable at the presently studied site. Irregular mowing of managed plots every year affects to a greater or lesser extent the population of premature *P. alcon* (when *G. pneumonanthe* is cut too early caterpillars are not able to finish the phytophagous part of their development) and it is difficult to distinguish natural cycles of abundance from anthropogenic factors. Moreover the analysis of the available orthophotomaps indicates that recent successional changes clearly reduced the *P. alcon* habitat at one of the plots of land, where just a single large oak was present for the past 15 years, and now scrubs and trees cover a significant part of the area. Other meadows were still open but encroachment of the invasive *Lupinus* was observed and goldenrods could be problematic in the future, taking into consideration that they were widespread in the vicinity. It is known that long term abandonment may lead to adverse changes in ant species composition in the habitats of *P. alcon* [71], and indeed we noticed that *M. scabrinodis* became less frequent in unmanaged patches in higher vegetation.

The site in the Białowieża Forest creates the potential for further studies, since it consists of distinct meadow stripes, and the suitability of managed plots changes from year to year due to differences in mowing. Studies of the effects of different management practices on particular parts of this unique system (butterflies, plants, ants and parasitoids) are vital in order to recommend optimum management.

Although our results suggest that the seasonal adult population of the parasitoid wasp is much more numerous than the host population in fact it may be even more at risk in case of habitat reduction [72]. Parasitoids are considered ultimate indicators of the biodiversity of *Phengaris (Maculinea*) systems, and their presence proves high habitat quality and the existence of large, functional metapopulations [73,74]. The presently studied site is highly isolated and there is little doubt that its relatively large area is a key factor in making it unique.

## 5. Conclusions

The investigated population is remarkable due to its exceptionally high density of adults and simultaneously high infestation rate by a specific parasitoid. We hope that the results of the present study will help to attract attention to the protection of this site, even though *P. alcon* is not a habitat directive species, and therefore is a secondary target of conservation in the Natura 2000 site Puszcza Białowieska.

## Figures and Tables

**Table 1:** Estimated adult population sizes (*N*) and densities derived from MRR studies of *Phengaris alcon* across Europe.

Ecotype of *Phengaris alcon*	Site	Country	Year(s)	*N*	Area (ha)	Density (*N*/ha)	Source
Higrophilous (*Gentiana pneumonanthe*)	Budy	Poland	2019	1460	1.71	854	present study
	Caselette	Italy	1997–2010	1600	2.9	52–552	[20,47]
	Mottarone	Italy	2009–2010	346–388	1.2	288–323	[47]
	Rauzes	France	2004–2007	281–1222	13	22–94	[48]
	Fanatele	Romania	2010	1313	40	33	[49]
	Bokiny	Poland	2015	40	5.47	7	[20]
Xerothermophilous (*Gentiana cruciata*)	Talesberg	Germany	1993	496	0.36	1378	[45]
	Rimetea	Romania	2012	699	0.93	752	[35]
	Schmandberg	Germany	1990	108	0.5	216	[50]
	Sengenberg	Germany	1990	262	1.5	175	[50]
	Wiedberg	Germany	1993	85	0.58	147	[45]
	Fanatele Domnesc	Romania	2010	1073	40	27	[49]
	Rieseler Berg	Germany	1990	38	2.25	17	[50]
